# Acid sphingomyelinase as a pathological and therapeutic target in neurological disorders: focus on Alzheimer’s disease

**DOI:** 10.1038/s12276-024-01176-4

**Published:** 2024-02-09

**Authors:** Byung Jo Choi, Min Hee Park, Hee Kyung Jin, Jae-sung Bae

**Affiliations:** 1 KNU Alzheimer’s Disease Research Institute, Kyungpook National University, Daegu, 41566 South Korea; 2 Department of Physiology, School of Medicine, Kyungpook National University, Daegu, 41944 South Korea; 3 Department of Laboratory Animal Medicine, College of Veterinary Medicine, Kyungpook National University, Daegu, 41566 South Korea

**Keywords:** Alzheimer's disease, Neural ageing

## Abstract

Over the past decade, numerous studies have highlighted the importance of acid sphingomyelinase (ASM) in disease treatment in humans. This enzyme functions primarily to generate ceramide, maintain the cellular membrane, and regulate cellular function. However, in the blood and brain of patients with neurological disorders, including major depression, ischemic stroke, amyotrophic lateral sclerosis, multiple sclerosis, and Alzheimer’s disease (AD), elevated ASM levels significantly suggest disease onset or progression. In these diseases, increased ASM is profoundly involved in neuronal death, abnormal autophagy, neuroinflammation, blood–brain barrier disruption, hippocampal neurogenesis loss, and immune cell dysfunction. Moreover, genetic and pharmacological inhibition of ASM can prevent or ameliorate various diseases. The therapeutic effects of ASM inhibition have prompted the urgent need to develop ASM inhibitors, and several ASM inhibitors have been identified. In this review, we summarize the current knowledge on the critical roles and mechanisms of ASM in brain cells and blood that are associated with different neuropathological features, especially those observed in AD. Furthermore, we elucidate the potential possibility and limitations of existing ASM-targeting drugs according to experimental studies in neurological disorder mouse models.

## Elevated ASM levels: a key indicator of neurological disease onset

Understanding how certain cell functions contribute to diseases is vital for creating treatments. This study explores sphingolipid metabolism. However, when this process malfunctions, it can cause various diseases. The enzyme acid sphingomyelinase is key as it produces ceramide, a lipid that, in excess, can cause cell death and contribute to diseases like Alzheimer’s and major depression. The study compiles recent findings on how ASM contributes to neurological disorders, particularly Alzheimer’s disease. It investigates how ASM impacts brain cells and blood, leading to symptoms like memory loss and inflammation. The review also discusses potential drugs that target ASM, offering hope for treating these disorders. The results show that high ASM levels in the brain and blood are linked to the development and progression of neurological disorders.

This summary was initially drafted using artificial intelligence, then revised and fact-checked by the author.

## Introduction

An important cellular pathway involved in cell division, survival, proliferation, immune response, and inflammation is sphingolipid metabolism^1–3^. Although sphingolipid mediators and their enzymes contribute to a small proportion of the total cellular lipid pool, their deficiency or accumulation may result in the pathogenesis of numerous human diseases^2–6^. Acid sphingomyelinase (ASM), a significant sphingolipid-metabolizing enzyme, plays a specific role in sphingomyelin hydrolysis into ceramide and phosphocholine^7^. As ASM-generated ceramide is involved in a variety of cellular processes, including cell death, apoptosis, differentiation, inflammation, and senescence, in various human diseases^8–12^, ASM has been suggested to be an important pathological target.

The sphingomyelin phosphodiesterase 1 (*SMPD1*) gene, the gene encoding ASM, is responsible for the generation of two distinct ASM types^7,13–15^. The lysosomal type is targeted to the endolysosomal compartment upon the processing of a common ASM precursor protein inside the Golgi, whereas the secreted type is released into the extracellular space^13–15^. In the lysosome, lysosomal ASM serves as a lipase for lipid digestion and helps regulate lysosomal membrane stabilization^14,16^. Lysosomal ASM genetic deficiency results in Niemann–Pick types A and B, which are lysosomal storage disorders characterized by progressive sphingomyelin accumulation and subsequent organ dysfunction^17^. The secretory role and function of ASM have not been well characterized compared to those of lysosomal ASM. However, pathological secretory ASM has recently been shown to be involved in various neurological disorders, particularly major depression, amyotrophic lateral sclerosis (ALS), Parkinson’s disease (PD), and Alzheimer’s disease (AD)^15,18–21^. In these diseases, abnormal ASM expression or activity in blood and brain cells induces apoptosis, increased permeability, abnormal autophagy, neuroinflammation, and immune cell dysfunction^22–26^, thereby contributing to disease onset and progression.

In this review, we underscore the progress made in recent years, specifically on the pathological roles and mechanisms of ASM in several neurological disorders, with a particular focus on AD. Moreover, we elucidate the potential of existing or promising ASM-targeting drugs to provide a basis for the prevention and treatment of neurological disorders.

## Pathological roles and mechanisms of ASM related to various neuropathological features

Neurological disorders negatively affect the function of the central nervous system (including the brain and spinal cord) and peripheral nervous system. These disorders can be triggered by genetic or congenital abnormalities, infection, environmental factors, brain damage, nerve injury, autoimmune dysfunction, or age-related degeneration^27–31^. Additionally, brain cell death, synaptic dysfunction, neuroinflammation, blood–brain barrier (BBB) disruption, and specific protein aggregation have been identified as common pathological features in neurological disorders^32–36^. Previous studies have demonstrated that high ASM expression or activity is associated with the induction and progression of these pathological features^15,18,19,21,24–26^. In this section, we elucidate how increased ASM in neurons, microglia, astrocytes, BBB endothelial cells (ECs), plasma, and blood immune cells contributes to neurological disorder development.

### Pathological effects of neuronal ASM

In neurological disorders, the pathological roles of neuronal ASM are associated with cell death. Previous studies using a stroke mouse model reported that transient focal cerebral ischemia-induced heightened neuronal ASM activity and ceramide levels in wild-type (WT) mice^37^. Elevated neuronal ASM/ceramide levels disrupt cellular calcium homeostasis and increase oxidative stress in neurons, thus contributing to ischemic neuronal death^37^. Moreover, the production of inflammatory cytokines, such as tumor necrosis factor-α, interleukin-1β (IL-1β), and interleukin-2, was also increased in the cortical tissue of ischemia-induced WT mice. Conversely, ASM knockout mouse neurons exhibited stable neuronal calcium homeostasis and decreased oxidative stress. No detectable increase in inflammatory cytokines in the cortex of ischemia-induced ASM knockout mice was found, leading to a significant reduction in neuronal cell death and infarct size, thereby improving behavioral deficits^37^. These results indicated that the ASM/ceramide pathway plays a major role in neuronal oxidative stress induction and inflammatory cytokine production through ischemia, suggesting the possible utility of therapeutics targeting this pathway for the treatment of patients with stroke. The effects of ASM-mediated neuronal cell death have also been reported in ALS and AD patients. High ASM or ceramide levels in the spinal cords of patients with ALS or in model mice result in neuromuscular junction and motor neuron loss, which are the primary pathologies of ALS^19,38^. Moreover, genetic ASM inhibition considerably attenuated motor behavioral dysfunction and spinal cord neuronal loss in an ALS mouse model^19^. Although the underlying molecular mechanism of ASM-mediated motor neuronal loss should be explored further in different ALS mouse models, this phenomenon could be correlated with neuronal apoptosis, defective neuronal autophagy, and abnormal protein accumulation in motor neurons. Several studies have shown high ASM and ceramide levels in the brains of patients with AD and in model mice^15,21,25,26,39–41^. Both amyloid beta (Aβ) oligomers) and hyperphosphorylated tau in the AD brain may increase ASM and ceramide levels in neurons^41^. Membrane remodeling or oxidation in neurons by ASM, ceramide, and lipid accumulation could in turn activate beta-secretase, leading to increased Aβ formation and ultimately neuronal death^42^.

In the hippocampus, the ASM/ceramide pathway is involved in neuronal proliferation and maturation. In in vitro and in vivo experiments using AD models, elevated ASM levels in hippocampal neural stem cells and neurons induced a significant decline in neuronal proliferation, survival, and neurogenesis through an increase in ceramide production^43^. These abnormalities contributed to hippocampal synaptic plasticity derangement and memory function impairment in AD. Additionally, previous studies revealed that the hippocampal ASM/ceramide pathway mediated the neurobiological and behavioral effects associated with major depression^44–46^. For example, ASM- or ceramide-overexpressing mice exhibited hippocampal neurogenesis impairment and decreased neuronal proliferation, maturation, and survival. These mice also exhibited depression-like behaviors, such as suppressed feeding, preference for dark spaces, and reduced grooming and mobility. ASM/ceramide pathway inhibition via genetic or pharmacological means normalizes these events^44–46^. In particular, several antidepressants increased hippocampal neurogenesis and reversed hippocampal atrophy associated with major depression through ASM/ceramide pathway inhibition. The negative effects of the ASM/ceramide pathway on hippocampal neurons could be associated with a decrease in Akt activation, which is vital for neuronal proliferation, reactive oxygen species formation, and proinflammatory cytokine production. Moreover, the pathogenesis of major depression is related to changes in cellular plasticity in the hippocampus and hippocampal atrophy^46–48^, indicating the crucial involvement of the ASM/ceramide pathway in major depression pathologies. Although the exact pathological mechanisms of this pathway in major depression are poorly understood, these observations indicate that ASM/ceramide acts as a negative regulator of hippocampal neurogenesis and neuronal maturation and survival in major depression.

**Fig. 1:**
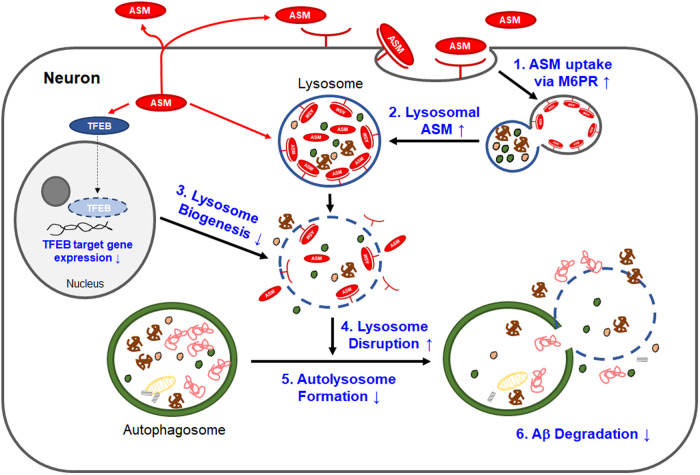
Neuronal acid sphingomyelinase (ASM) mediates autophagic dysfunction in the Alzheimer’s disease (AD) brain. In AD, neuronal ASM is increased by environmental or cellular stressors. Intracellular and secreted ASM undergoes lysosomal uptake via M6PR. Excessively increased lysosomal ASM affects lysosomal disruption, and intracellular ASM decreases lysosomal biogenesis by reducing the expression levels of the transcription factor EB target gene in the cell nucleus. This lysosomal disruption by ASM leads to the inhibition of autophagic protein degradation and subsequent autophagosome accumulation, as indicated by the expression of abnormal proteins such as Aβ and other cytotoxic proteins. Eventually, autolysosome formation decreases, and autolysosome deposition contributes to Aβ deposition in the AD brain.

In the mammalian nervous system, particularly in neurons, autophagy plays a central role in maintaining neuronal health and protein homeostasis through the removal of large and insoluble protein aggregates^49–51^. Moreover, in the AD brain, autophagy dysfunction is implicated in Aβ accumulation. The high neuronal ASM levels observed in patients with AD and model mice disrupt the normal autophagy pathway. A previous study revealed the association between increased neuronal ASM and autophagy dysfunction^25^. In AD neurons, ASM in lysosomes is primarily increased. Additionally, extracellularly secreted ASM enters lysosomes via the mannose 6-phosphate receptor in the cell membrane^25^. Increased lysosomal ASM decreased lysosomal biogenesis through the downregulation of transcription factor EB target genes in the nuclear compartment of neurons, leading to increased lysosomal disruption. Thus, the reduction in autolysosome formation was attributed to a decrease in lysosomes, leading to an impairment of autophagic Aβ degradation^25^ (Fig. 1). Although a previous study established that neuronal ASM plays a critical role in autophagic degradation, the relevance of ceramides in this type of autophagic dysfunction has been poorly explored. Several studies have reported increased ceramide levels in AD, and ceramide is also known to be involved in the autophagy pathway. For example, ceramide can induce autophagy-mediated cell death through the transcriptional upregulation of Beclin1 expression^52,53^, which acts during the initiation stage of autophagy^54,55^. Ceramide might also affect neuronal cell death in the AD brain. However, since ASM does not directly affect Beclin1 expression, as shown in a previous study^25^, ASM and ceramide are deemed to have independent negative effects on autophagic dysfunction in the AD brain. From this perspective, neuronal ASM/ceramide pathway inhibition is expected to have beneficial effects on decreasing Aβ accumulation and neuronal cell death by normalizing autophagic dysfunction in the AD brain.

### Pathological effects of ASM on microglia and astrocytes

In the CNS, microglia are the major immune cells. Microglia play vital roles in neuroinflammation and in neuronal survival and death. The microglial inflammasome, one of the major drivers of neuroinflammation, is an important component of the immune response to tissue damage, and inflammasome dysregulation has been implicated in various neurological disorders^56–58^. A recent study reported that ASM is involved in the formation of the NLR family pyrin domain containing 3 (NLRP3) inflammasome^59^. Elevated ASM concentrations resulted in NLRP3/ASC/caspase-1 inflammasome complex enhancement, caspase-1 activation, and IL-1β and IL-18 generation through ceramide production. An increase in the levels of these proinflammatory proteins ultimately leads to neuroinflammation. Conversely, pharmacological ASM inhibition markedly attenuated these events in mice with brain injury^60^. Although further studies are warranted to elucidate the exact molecular mechanism through which ASM regulates inflammasome signaling in microglia, previous studies have shown that ASM may be an essential factor for the activation of the microglial NLRP3 inflammasome complex in the neurological inflammatory cascade.

The pathological effects of ASM on astrocytes have been investigated in multiple sclerosis (MS)^61^, a chronic inflammatory disease characterized by excessive immune cell infiltration into the CNS, axonal damage, demyelination, and astrogliosis. Astrocytes isolated from the brain tissues of patients with MS showed increased mRNA expression of ASM and ceramide production, which induced proinflammatory cytokine release and neuroinflammatory event promotion. Furthermore, increased levels of ASM and ceramide, which are found in astrocyte end feet covering the brain vasculature, directly affected BBB function and increased the trans-endothelial migration of peripheral immune cells in vitro. Pharmacological inhibition of ASM in reactive human astrocytes reduced ceramide production, thereby attenuating neuroinflammation and neurovascular disruption. In astrocytes in the rat hippocampus, elevated ASM and ceramide levels were also detected after cerebral ischemia induction^62^. Ceramide accumulation in astrocytes induced Jun-N-terminal kinase (JNK) and protein phosphatase (PP) 2A activation, which is associated with neuronal damage in cerebral ischemia. Thus, these findings suggest that astrocyte-derived ASM is a mediator involved in the neuropathological features of MS and cerebral ischemia.

### Pathological effects of brain EC-derived ASM

The prevalence of most neurological disorders increases markedly with age, suggesting that aging is one of the risk factors for neurological disorders. A previous study showed that the tissue-specific contribution of ASM activity was greater in the brain tissues of 20-month-old mice than in the tissues of other organs, such as the liver, kidney, spleen, heart, lung, stomach, and genitals, as well as muscle and fat, in young mice^24^. The considerable increase in ASM levels in the brains of old mice was associated with microvessels. More specifically, the ECs that make up the BBB were the primary contributors to increased ASM activity in the brains of old mice^24^. An increase in brain EC-derived ASM levels induced apoptosis via p53 signaling activation. The brain vessels of old mice or ASM-treated brain ECs exhibited an upregulation of proapoptotic signaling proteins, such as p53 and Bax, and a downregulation of antiapoptotic signaling proteins, such as Bcl-2, without ceramide level changes^24^. This observation indicates that increased brain EC-derived ASM concentrations directly affect endothelial apoptosis in old mice.

**Fig. 2:**
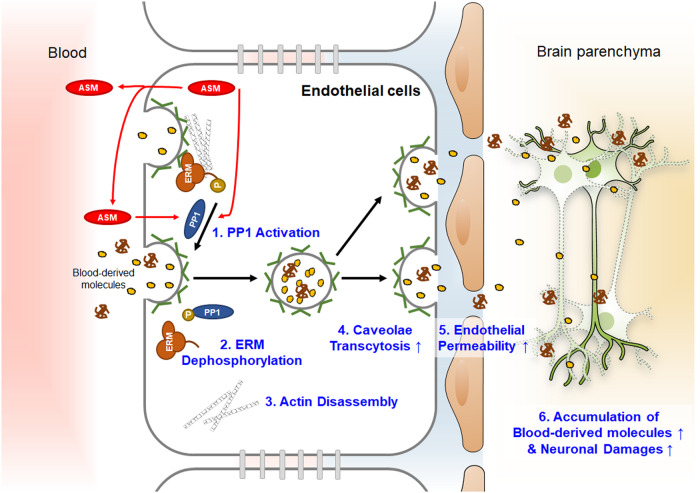
Brain endothelial cell-derived ASM mediates blood–brain barrier (BBB) disruption in the aged brain. In the aged brain, ASM activity is strongly increased in the endothelial cells that make up the BBB. Brain endothelial ASM induces PP1-mediated ezrin/radixin/moesin dephosphorylation via autocrine and/or paracrine effects and further causes cytoskeletal disassembly and excessive caveolae internalization. Increased caveolae-mediated transcytosis by ASM results in BBB hyperpermeability and blood-derived molecule extravasation into the brain parenchyma, ultimately leading to neuronal cell death.

BBB leakage is caused by BBB cell death, tight junction damage, or excessive transcytosis and may lead to abnormal protein accumulation, neuroinflammation, and neuronal damage in the brain. Thus, one of the therapeutic strategies for treating aging or neurological disorders is inhibiting BBB breakdown. Brain EC-derived ASM in old mice was associated with increased caveolae-mediated endothelial transcytosis and cell apoptosis, promoting BBB hyperpermeability^24^; conversely, it had no effect on tight junction damage, which is another probable cause of BBB leakage. A high ASM concentration in brain ECs induced intracellular actin cytoskeleton disassembly through an increase in ezrin/radixin/moesin (ERM) protein dephosphorylation via protein phosphatase 1 (PP1) activation^24^. Additionally, it resulted in actin filament loss, which prevented caveolae internalization in brain ECs^63–67^, contributing to BBB hyperpermeability in old mice (Fig. 2). The systematic gain-of-function of brain EC-derived ASM on BBB integrity has been well demonstrated in conditional transgenic mice with brain EC-specific ASM overexpression^24^. Despite being only 13 months old, these mice showed significantly increased ASM activity in brain microvessels, vessel density reduction, and increased BBB leakage, all of which were similar to the observations in 20-month-old WT mice. Moreover, caveolae-mediated transcytosis and ERM dephosphorylation were observed in the brain microvessels of these mice. Notably, these mice exhibited reduced neuronal cells and severe memory impairment. Specifically, the inhibition of BBB EC-derived ASM using *Smpd1*-miR RNA interference (RNAi) improved BBB leakage and neuronal dysfunction in brain EC-specific ASM-overexpressing mice and old mice. These findings suggest new avenues for understanding the mechanisms of ASM-mediated BBB disruption and, thereby neurological brain pathologies in aging. Until now, BBB dysfunction as a cause or sequela of neurodegeneration has remained a subject of active debate. Although additional research is warranted to address this topic, previous findings provide sufficient data indicating that BBB destruction may be a cause of neurodegeneration.

In MS, brain EC-derived ASM regulates adhesion molecules in the vasculature, such as intercellular adhesion molecule-1 (ICAM-1) in the endothelial plasma membrane, promoting T lymphocyte migration into the brain^68^. Specifically, ceramide generated by ASM facilitated ICAM-1 clustering in the brain endothelial plasma membrane through the regulation of its interaction with filamin and ezrin phosphorylation. Consequently, elevated ICAM-1 levels induced T-cell adhesion and transmigration in the brain endothelium, further exacerbating brain damage. However, in ASM-deficient brain ECs, these events were less prominent^68,69^, indicating potential therapeutic strategies targeting brain EC-derived ASM for preventing MS lesion formation. In other neurological disorders, many speculations have emerged regarding the association between brain EC-derived ASM and pathological features. This is because ASM is more abundant in the brain than in other tissues, and ECs, in particular, are a rich source of ASM. Thus, to develop new therapeutic interventions for neurological disorders, further studies that more clearly elucidate the pathological effects of brain EC-derived ASM are warranted.

### Pathological effects of ASM in the blood

For decades, in various neurological disorders, several studies have reported increased blood ASM levels. Previous research has demonstrated that plasma ASM activity is significantly increased in individuals with AD compared with that in healthy-aged individuals but not in PD^24^. In particular, plasma ASM activity in patients with AD was shown to be positively correlated with disease progression^26,43^, indicating the critical role of ASM as a potential biomarker for AD. In patients with MS, ASM activity was elevated in blood samples. Although no correlation has been reported between disease progression or clinical score^70^, high blood ASM activity may be related to peripheral pathological features such as immune cell dysregulation and inflammation in MS patients. In the plasma of ALS patients with *FUS*, *SOD1*, or *TBK1* mutations, increased ASM activity was also observed. These gene mutations affect RNA processing and lead to oxidative stress, cytoskeletal instability, protein aggregation and degradation, and autophagy dysfunction^71–73^. As ASM can increase during various pathological processes or environmental stresses, mutations in these genes may be closely associated with increased ASM in ALS patients. Despite the importance of blood ASM as a biomarker, most studies on neurological disorder pathogenesis have focused on CNS tissues, and the effects on the peripheral nervous system regarding disease onset and progression have not been fully elucidated. For major depression, ASM activity is high in the blood mononuclear cells of patients with severe major depression, and ASM activity is correlated with disease severity^18,74^. Further exploration of the pathophysiological significance of increased ASM activity in the blood mononuclear cells of patients with major depression is needed. However, this is deemed to be related to the increase in inflammation observed in major depression, as ASM mediates inflammation via ceramide production^59,60^. This hypothesis is supported by previous studies showing that increased ASM in macrophages promotes inflammatory signals and cytokine production^75,76^. Proinflammatory cytokines present in the blood at high levels promote BBB disruption and enter the brain, leading to neuroinflammation and neuronal death^77–79^. Thus, elevated ASM in the blood is expected to be significantly involved in neurological pathologies.

**Fig. 3:**
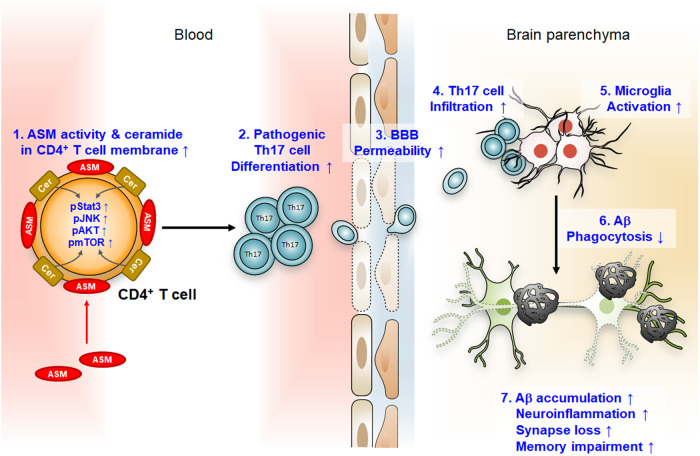
Blood ASM mediates the promotion of various neuropathological features in AD. Increased blood ASM is recruited to CD4^+^ T-cell membranes and induces ceramide level elevation. The Stat3, JNK, and mTOR signaling phosphorylation induced by ceramide promotes pathogenic Th17 cell differentiation from CD4^+^ T cells. Cytokines secreted from pathogenic Th17 cells lead to BBB disruption through a reduction in tight junction protein expression, and increased BBB permeability facilitates the entry of these cells into the brain parenchyma. Pathogenic Th17 cell infiltration affects microglial activation and leads to defective phagocytic functions, consequently accelerating Aβ accumulation, neuroinflammation, synaptic loss, and memory impairment.

Recently, it has been shown that the critical role and mechanism of blood ASM are associated with AD brain pathologies^26^. A research group first revealed that ASM activity in the blood increased with disease progression or age in patients with AD and model mice without changes in blood ceramide levels. In vivo experiments employing surgical parabiosis revealed accelerated neuropathological features, including increased Aβ deposition and neuroinflammation, in young AD mice exposed to the blood of ASM-overexpressing mice. Increased blood ASM was recruited to CD4^+^ T-cell membranes and led to cell apoptosis and pathogenic T helper 17 (Th17) cell differentiation through ceramide production in the cell membrane. The ASM/ceramide pathway in CD4^+^ T-cell membranes stimulated key signals involved in Th17 cell differentiation, such as Stat3, JNK, AKT, and mTOR phosphorylation, and upregulated the expression of pathogenic Th17 cell genes and cytokines. Cytokines secreted by pathogenic Th17 cells reduced tight-junction protein expression in BBB-ECs and contributed to increased BBB permeability and Th17 cell infiltration from the blood into the brain. Pathogenic Th17 cells that directly infiltrated into the brain exacerbated the inflammation and defective Aβ phagocytic function of the microglia, thus accelerating neuroinflammation and Aβ accumulation in young AD mice (Fig. 3). Conversely, surgical parabiosis of 9-month-old AD mice and ASM knockout mice led to brain pathology prevention in AD mice. Blood exchange between these mice led to elevated levels of ASM antibodies in the blood of AD mice, and it significantly reduced blood ASM activity but not in the brain. Specific inhibition of blood ASM activity prevented changes in immune cell populations, BBB disruption, neuroinflammation, Aβ deposition, and synaptic loss through a decrease in pathogenic Th17 cells in the blood and brain of AD mice. Moreover, ASM peptide immunization exerted protective effects against these pathological features and memory impairment in AD mice. These findings indicate that targeting blood ASM is a promising therapeutic strategy for the prevention of various neuropathological features of AD.

In patients with and mouse models of neurological disorders, such as major depression, MS, ALS, and AD, an increase in Th17 cells has been observed^80–84^. Moreover, alterations in other immune cells, including neutrophils, monocytes, and macrophages, have been detected in these diseases. Additionally, increased blood ASM levels were also found in these disorders, indicating a positive correlation between immune cell alterations and blood ASM in terms of pathology. Although an increase in blood ASM directly affected the Th17 cell population, blood ASM reduction effectively normalized the increase in neutrophils, proinflammatory monocytes, and M1 macrophages by reducing the number of pathogenic Th17 cells in the blood and brain in an AD mouse model^26^. These results are likely related to the fact that pathogenic Th17 cells can induce inflammatory responses or chemotaxis in other immune cells, such as monocytes, macrophages, and neutrophils^84–86^. Thus, blood ASM reduction may contribute to the improvement of pathologies through the restoration of normal Th17 cell numbers and even other immune cell alterations in these neurological disorders. Although an investigation of the pathological roles of blood ASM-mediated immune cell dysregulation in other neurological disorders is warranted, a therapeutic strategy targeting blood ASM could be helpful in the prevention and treatment of neurological disorders.

## The potential and limitations of ASM-targeting drugs for neurological disorders

Many research groups have attempted to develop ASM inhibitors, as ASM inhibition has been shown to be vital in various neurological disorders. Recently, several functional and direct ASM inhibitors have been identified, and their therapeutic effects have been observed in mouse models of neurological disorders. In the following sections, the beneficial effects and limitations of functional and direct ASM inhibitors in the treatment of neurological disorders will be reviewed.

### Functional inhibitors of ASM (FIASMAs)

FIASMAs functionally inhibit lysosomal ASM activity in a reversible and additive manner. As small molecules, FIASMAs can easily enter the inner leaf of lysosomal membranes, and their weak bases cause ASM to detach from lysosomal membranes, resulting in ASM degradation within the lysosome. These inhibitors include several antidepressant drugs and other cationic amphiphilic drugs that are approved for medical use in humans and have minimal toxicity^21,87–91^. Thus, these compounds are considered promising drugs for treating neurological disorders caused by increased ASM activity. In this regard, previous studies have revealed the ASM-mediated therapeutic effects of several antidepressant drugs, such as amitriptyline, fluoxetine, and imipramine, in animal models of neurological disorders (Table 1). Amitriptyline and fluoxetine reduced ASM activity and ceramide levels in the brains of major depression model mice^44,92^, contributing to neuronal loss and behavioral dysfunction improvement. The decreased ASM activity caused by amitriptyline and fluoxetine also promoted a reduction in leukocyte brain infiltration, angiogenesis, neuronal survival, and motor coordination recovery in an ischemic stroke mouse model^93^. Moreover, long-term amitriptyline treatment in aged or AD model mice attenuated BBB disruption, neuronal loss, abnormal neuronal autophagy, Aβ accumulation, and memory impairments^24,25,43^. In these mice, amitriptyline reduced ASM activity in the blood and brain. This observation is speculated to be an indirect effect of reduced ASM secretion in the brain. In a rat model of traumatic brain injury (TBI), imipramine reduced hippocampal neuronal death, neuroinflammation, neurological deficits, and cognitive dysfunction through the restoration of the TBI-induced overexpression of ASM and ceramide^94^. Overall, most functional ASM inhibitors decrease both ASM activity and ceramide levels in the brains of major depression, ischemic stroke, TBI, aging, or AD model mice. The inhibition of the ASM/ceramide pathway by functional ASM inhibitors has beneficial effects on improving neuronal death, neuroinflammation, neurological deficits, and behavioral dysfunction in these neurological disorders.

**Table 1:** Therapeutic effects of functional inhibitors targeting ASM in neurological pathologies.

Name	Target tissue or cell	Disease	Mouse model	Therapeutic effects	Ref
Amitriptyline	Brain	Major depression	ASM-transgenic mice Stress-induced mice	Hippocampal neuronal loss ↓ Hippocampal neurogenesis ↑ Behavioral dysfunction ↓	^44,92^
	Brain	Ischemic stroke	Focal cerebral ischemia-induced mice	Leukocyte brain infiltration ↓ Angiogenesis ↑ Neuronal survival ↑	^93^
	Brain Blood	Alzheimer’s disease	APP/PS1	Aβ accumulation ↓ Abnormal neuronal autophagy ↓ Memory impairment ↓	^25,43^
	BBB-ECs Blood	Aging	20-month-old WT mice	BBB disruption ↓ Neuronal loss ↓ Memory impairment ↓	^24^
Fluoxetine	Brain	Major depression	ASM-transgenic mice Stress-induced mice	Hippocampal neuronal loss ↓ Hippocampal neurogenesis ↑ Behavioral dysfunction ↓	^44,92^
	Brain	Ischemic stroke	Focal cerebral ischemia-induced mice	Angiogenesis ↑ Motor-coordination recovery ↑	^93^
Imipramine	Brain	Traumatic brain injury	TBI-induced rat	Hippocampal neuronal death ↓ Neuroinflammation ↓ Cognitive dysfunction ↓	^94^

To improve neurological pathology, FIASMAs must penetrate through biological membranes to achieve their inhibitory effects, similar to how substances must cross the BBB. Therefore, most FIASMAs can efficiently cross the BBB. Additionally, because several functional inhibitors are used in the long term and their side-effect profiles are well known, FIASMAs may become easily accessible medications for patients with neurological disorders. However, these functional inhibitors lack specificity, and their off-target effects and exact molecular mechanisms in neurological disorders have not yet been characterized. Thus, further studies to develop more specific and effective ASM inhibitors may be helpful in the clinical treatment of patients with these disorders.

### Direct inhibitors of ASM

To overcome the limitations of FIASMAs, several direct ASM inhibitors, such as arc39, ad2765, and ent-12, have been identified^95–98^. These compounds act on ASM with high potency in cells; however, in vivo, their efficacy remains unclear. The pharmacological properties and in vivo efficacy of a recently discovered direct inhibitor of ASM, KARI 201, have been demonstrated, especially in AD mouse models^43^. This small compound demonstrated a competitive mode of inhibition versus sphingomyelin by binding at ASM active sites, leading to the inhibition of ASM activity and ceramide production. Moreover, KARI 201 selectively inhibited ASM activity with no substantial off-target effects on other enzymes or sphingolipids. The pharmacokinetic results also suggested excellent brain distribution of KARI 201. In in vivo experiments, KARI 201 had a considerably greater inhibitory effect on ASM in the blood and brain of AD mice than did amitriptyline despite the short administration period and low dose. In particular, KARI 201 blocked neuronal ASM activity and reduced ceramide levels, contributing to the rescue of various neuropathological features in the brains of AD mice, including Aβ deposition, neuronal autophagic dysfunction, neuroinflammation, synaptic loss, decreased hippocampal neurogenesis and synaptic plasticity, and memory impairment (Table 2). The efficacy of KARI 201 in terms of improving these neuropathological features was superior to that of amitriptyline. Unexpectedly, KARI 201 has been found to act as a ghrelin receptor agonist that exerts synergetic effects on improving hippocampal neurogenesis and memory impairment in AD mice. A previous study has shown the therapeutic roles and mechanisms of KARI 201 focused on brain pathologies in AD mice^43^. However, direct ASM inhibitors, including KARI 201, are expected to have systemic beneficial effects on BBB damage, pathogenic Th17 cells, and immune cell dysregulation in AD^21,43,99^. Thus, future studies exploring the therapeutic effects of direct ASM inhibitors on ASM-mediated systemic pathological features may provide additional concrete evidence that a direct ASM inhibitor could be a promising drug for treating numerous diseases characterized by increased ASM activity.

**Table 2:** Therapeutic effects of direct inhibitors targeting ASM in neurological pathologies.

Name	Target tissue or cell	Disease	Mouse model	Therapeutic effect	Ref
ARC 39 AD 2765 *ent*-12	Not proven				
KARI 201	Neurons Blood	Alzheimer’s disease	APP/PS1 5xFAD	Aβ accumulation ↓ Abnormal neuronal autophagy ↓ Neuroinflammation ↓ Synaptic loss ↓ Hippocampal neurogenesis ↑ Synaptic plasticity ↑ Memory impairment ↓	^43^
23A12C3	CD4^+^ T cells Blood	Alzheimer’s disease	APP/PS1	Pathogenic Th17 cells ↓ Aβ accumulation ↓ Neuroinflammation ↓ Memory impairment ↓	^26^

A recent study elucidating the critical role of blood ASM in relation to various AD pathologies indicated the potential of immunotherapy for targeting blood ASM for AD prevention^26^. Based on this possibility, the newly generated monoclonal ASM antibody 23A12C3 displayed high ASM inhibitory potency and selective binding to ASM^26^. Moreover, this antibody efficiently decreased the blood ASM activity of AD mice, resulting in the inhibition of ASM activity and the accumulation of various kinds of ceramide in the CD4^+^ T-cell membrane. Consequently, 23A12C3 led to a reduction in pathogenic Th17 cells in the blood and brain and exerted prophylactic effects on neuroinflammation, Aβ accumulation, and memory impairment (Table 2). High target accuracy and specificity, reduced off-target effects, and a known pharmacokinetic profile are among the features of the antibody-based immunotherapeutic approach; however, this approach also has disadvantages, such as an unexpected immune response and high cost for designing, generating, and humanizing antibodies. Nevertheless, immunotherapy targeting specific pathological factors has emerged as a promising approach for treating neurological disorders because existing drugs aid only in managing or delaying symptoms. In this regard, some of the Aβ-targeting antibodies developed for AD treatment have been shown to reduce the load of amyloid and inhibit cognitive decline in a clinical study^100–102^. However, further investigation is needed for the prevention of side effects and BBB penetration require. Thus, many researchers have attempted to develop an antibody to improve BBB penetration as well as antibody specificity and affinity. Unlike Aβ-targeting antibodies, blood ASM-targeting immunotherapy exerted significant therapeutic effects on an AD mouse model despite the absence of BBB penetration. Although further preclinical and clinical studies are warranted in the future, this approach is anticipated to constitute a next-generation immunotherapy for AD and even other neurological disorders.

## Concluding remarks

**Fig. 4:**
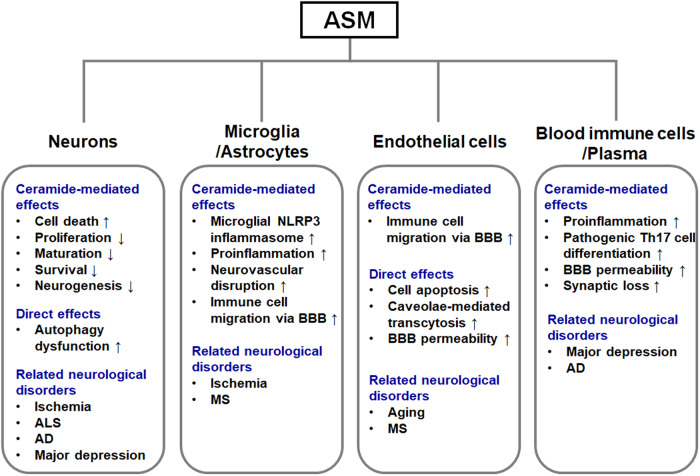
Pathological effects of elevated ASM in brain cells or blood from patients with various neurological disorders. ALS amyotrophic lateral sclerosis, AD Alzheimer’s disease, MS multiple sclerosis.

In this paper, the pivotal roles and pathological mechanisms of ASM in neurological disorders were summarized (Fig. 4). An increased ASM level in brain cells and the blood was found to be involved in the onset or progression of various neuropathological diseases. However, because the specific pathological roles of ASM have been demonstrated in only a few diseases, especially AD, further research on other neurological disorders is warranted. ASM is also activated and increased in response to various stressors, such as inflammation, infection, and harmful environmental factors, and can lead to systemic diseases. Additionally, elevated ASM levels are observed in severe inflammatory diseases, such as hepatitis C infection, sepsis, systemic inflammatory response syndrome, inflammatory bowel disease, systemic vasculitis, and lymphohistiocytosis^15^. Thus, future extensive studies on ASM-mediated systemic pathologies are needed to broaden the scope of the disease applications of ASM-targeting drugs.
